# Improved Flutter Ablation Outcomes Using a 10mm-tip Ablation Catheter

**Published:** 2010-12-26

**Authors:** Tiago Luiz Luz Leiria, Giuliano Becker, Teresa Kus, Vidal Essebag, Tomy Hadjis, Marcio Lerch Sturmer

**Affiliations:** 1 Servico de Eletrofisiologia do Instituto de Cardiologia do Rio Grande do Sul, Brazil; 2 Service d'Arythmie de l'Hopital du Sacre-Cœur de Montreal, Montreal, Canada

**Keywords:** Ablation, Electrophysiology, Clinical, Atrial Flutter

## Abstract

**Introduction:**

Radiofrequency(RF) ablation has become the first line of therapy for atrial flutter(AFL). Advances in catheter and mapping technologies have led to better understanding and different approaches for treating this arrhythmia. We describe the results of different approaches to ablate this arrhythmia.

**Materials and Methods:**

A cohort of 198 patients with isthmus dependent AFL. The techniques used were: 10mm-tip catheter with  power set to 100w, 8mm-tip catheter with power set to 60W and irrigated tip catheter.

**Results:**

212 procedures, including redos were done in 198 consecutive patients. We used irrigated tip catheters in 14 procedures, 8mm-tip in 55 procedures, and 10mm-tip in 143 procedures. Bidirectional block was achieved in 97.6% of cases with all techniques, with no difference among them. Procedure time was shorter in the 10mm-tip versus 8mm-tip(69.6±30.6min vs.105±43min) or irrigated tip(180±90min) (P<0.05). Fluoroscopy time was also shorter in the 10mm-tip versus  8mm-tip (24±18min vs. 37±23min) or irrigated tip (110±25min)(P<0.05). The cumulative incidence of failure during follow-up was 1.2%/year in the 10mm, 10.1%year in the 8mm and 6.9%year in the irrigated tip. The survival free of a new procedure was significantly higher among 10mm patients.

**Conclusions:**

In our series we found a high rate of acute success with the use of different techniques for AFL ablation. Procedure and fluoroscopic times were shorter with the use of 10mm-tip as compared with the others techniques. The long-term risk of recurrence was lower when we used the 10mm-tip catheter and the survival free of a second procedure was higher among patients treated with this catheter.

## Introduction

Isthmus-dependent right atrial flutter (AFL) is a well known arrhythmia commonly encountered in electrophysiology (EP) laboratories. Its electrophysiological mechanism is well established [1] and,  it can be cured by creating a line of bidirectional block from the tricuspid valve to the inferior vena cava in the region referred to as the cavo-tricuspid isthmus (CTI) [2]. Ablation of the CTI prevents the occurrence of the macro reentry that is responsible for AFL maintenance, and demonstration of bidirectional conduction block across the CTI after ablation is related to a long-term success (freedom from AFL recurrence) of around 90% [3].

In recent years, several catheter types and forms of energy have been used to create the CTI ablation line [3-9]. The most common form of energy used for this purpose is radiofrequency (RF). Adequate delivery of RF to the target region inside the heart requires a good interface of contact between the ablating electrode and the endocardial surface to ensure adequate power delivery in the target area. Sometimes, the anatomy of the CTI is highly variable with different configurations and topologies making the catheter ablation a difficult task [10]. To minimize these problems, different companies developed larger catheter tips (8mm-tip and 10-mm-tip) to improve tissue contact and to use high power output (70-100W), as well as irrigated catheters to maximize energy delivery by actively cooling the catheter tip.

Randomized, prospective studies have shown that large-tip electrode catheters or irrigated catheters are more effective than conventional 4-mm-tip catheters for AFL ablation [5,11]. There is also a single study that compared large 8-mm-tip versus 10-mm-tip showing that results are at least comparable with both techniques [12]. In this article we present the results of a registry of AFL ablations comparing three different techniques using irrigated tip, 8-mm-tip and 10-mm-tip catheters.

## Materials and Methods

### Patients

This is a cohort of 198 consecutive patients (79% males; mean age 61.5±13.6 years) referred to our institution for 'de novo' ablation of typical AFL between March 2003 until December 2008. Patients with prior AFL ablation (i.e. repeat procedure) were excluded from cohort entry.

### Procedure Description

Patients were brought to the EP lab in a fasting state. Femoral venous access was obtained under local anesthesia. A decapolar catheter was advanced into the coronary sinus (CS) and a second decapolar catheter was advanced to the antero-lateral right atrial (ALRA) position.

One of the following three commercially available ablation catheters were used for the procedure: 1) Navistar Thermocool® 3.5mm irrigated tip  - Biosense Webster inc.; 2) Navistar DS 8mm tip Biosense Webster inc. and 3) Blazer-II Xp 10mm tip EPT inc. The ablation catheter was advanced to the CTI where RF delivery was performed at the 6 o'clock position, using either an interrupted or continuous drag technique from the tricuspid annulus to the inferior vena cava, for up to 120 seconds, with a maximum target temperature of 60-65ºC. For ablation with a 10mm catheter the radiofrequency power was set to 100 watts, and to 60 watts for the 8mm-tip and to a maximum of 50W for the irrigated catheters.

The presence or absence of bidirectional isthmus conduction block was assessed during pacing from the coronary sinus ostium and low lateral right atrium at a cycle length 600 ms while performing RF delivery over the line. Ablation was repeated until isthmus block was achieved. Bidirectional isthmus block was confirmed by demonstrating a fully descending wavefront of activation in the contra lateral atrial wall during pacing from the coronary sinus ostium and low lateral right atrium, respectively. We also searched for split potentials (>100msec) along the ablation line. All electrograms were recorded with a commercial digital acquisition system (Prucka Engineering, Inc). For RF delivery with irrigated and the 8mm catheter we used a Stockert 70 RF Generator (Biosense Webster Inc) and for the 10mm a generator EPT-1000, EP Technologies, Inc.

### Outcomes

We defined acute success as being the achievement of bidirectional block by ablation as earlier explained. Patients with AFL recurrence during follow-up underwent a second  procedure.

### Statistical Analysis

Continuous data such as procedure duration, total fluoroscopy time, months of follow up, age, size of the left atrium, and ejection fraction were analyzed with the ANOVA test. Post-hoc analyses were done using Student-Newman-Keuls test for all pairwise comparisons. For the categorical variables we used the chi square test. Cumulative late recurrence was analyzed with Cox proportional hazards regression. The data bank, statistical calculations and graphs were performed using the software MedCalc® V.7.3.

## Results

A total of 198 patients underwent first time AFL ablation. A total of 212 procedures were done (including redo cases). Irrigated-tip was used as the ablation catheter in 14 procedures, 8mm-tip were used in 55 procedures, and 10mm-tip were used in 143 procedures. The mean follow-up time was 19±13 months. The clinical characteristics of all patients are demonstrated in Table 1.

The acute success defined as bidirectional block was achieved in 97.6% of cases with all three catheter types, with no difference among them. There was no difference in procedure success among the different operators (P=0.9).

**Figure 1:**
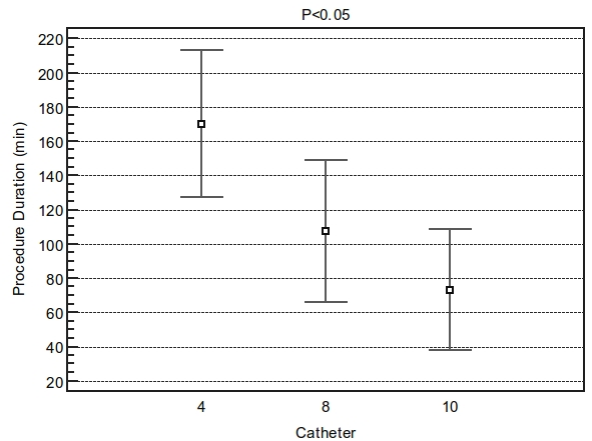
Difference in procedure duration among groups. ANOVA F-ratio: 71.11 Significance level:  P<0.001. The procedure time was significant shorter in the 10mm tip (69.6±30.6min) as compared to 8mm tip (105±43min) or irrigated tip (180±90min) Student-Newman-Keuls test for all pairwise comparisons with P<0.05. Catheter 4= irrigated tip.

**Figure 2:**
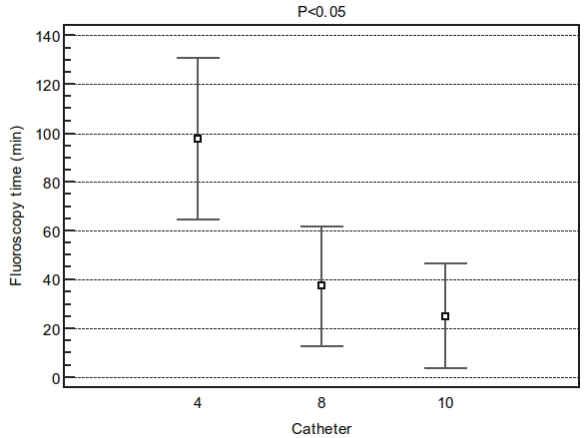
Difference in fluoroscopy time among groups. ANOVA F-ratio: 71.11 Significance level:  P<0.001. Fluoroscopy time was also shorter in the 10mm tip (24±18min) as compared to 8mm tip (37±23min) or irrigated tip (110±25min) Student-Newman-Keuls test for all pairwise comparisons with P<0.05. Catheter 4= irrigated tip.

The procedure time was significant shorter in the 10mm-tip as compared to 8mm-tip  or irrigated tip (P<0.05)(Figure 1). Fluoroscopy time was also shorter in the 10mm-tip as compared to 8mm-tip or irrigated-tip (P<0.05)(Figure 2).

**Figure 3:**
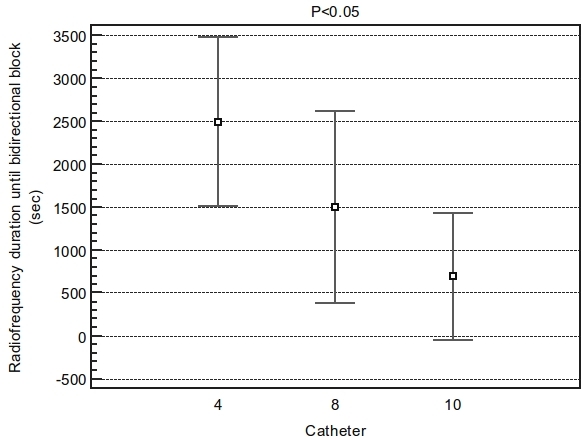
Difference in duration of radiofrequency use until bidirectional block among groups. ANOVA F-ratio: 37.95 Significance level:  P<0.001. Radiofrequency use was shorter in the 10mm tip (694sec) as compared to 8mm tip (1500sec) or irrigated tip (2500sec). Student-Newman-Keuls test for all pairwise comparisons with P<0.05. Catheter 4= irrigated tip.

The amount of radiofrequency delivery, to achieve bidirectional isthmus block, during the procedure (in seconds) was, as well, shorter in the 10mm-tip as compared to 8mm-tip or irrigated-tip (P<0.05)(Figure 3).

A total of 18 cases needed a second procedure. The cumulative incidence of failure (defined as a new procedure) during follow-up was: 1.2%/year in the 10mm;  6.9%/year in the irrigated tip and 10.1%/year in the 8mm. The relative risk for recurrence was 15.6(95%CI=3.6-67) when using the 8mm versus 10mm and 20.42 when using the irrigated compared with the 10mm (95%CI=4-101). Recurrences with the irrigated tip were done using the 8mm-tip catheter and those occurred after ablation with a 8mm-tip catheter were treated with a 10mm-tip.

**Figure 4:**
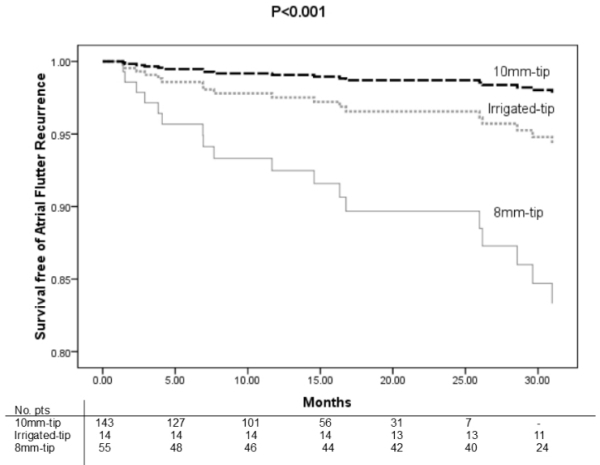
Cumulative survival free of flutter recurrence between different catheters using a proportion hazard model adjusted by age, sex, fluoroscopy time and medical operator.

Using a Cox regression analysis we were able to identify that the survival free of a new procedure was significantly higher among 10mm patients (Figure 4).

## Discussion

Several studies 5-8 in the literature have compared the use of 8mm tip catheter with irrigated tip  but only a few used the 10mm catheter against the 8mm-tip [12]. Comparisons among the three types of catheter are scarce. In our study we reveal, in a real world scenario, that the use of a large tip catheter with a high output power is more effective regarding procedure related endpoints and clinical outcomes in patients with isthmus dependent AFL.

It is important to mention that the acute success rate, defined as bidirectional block, during the ablation procedure was about the same with all techniques. This finding is also reported in other papers that compared different catheters for AFL ablation.

The duration of the procedure, total fluoroscopy and radiofrequency delivery times were significant shorter with the use of 10mm catheter and high power when compared with the other catheters. This finding remained true even when we controlled for the use of 3D mapping and other potential confounders. Feld et al.[12] showed similar results when comparing the use of 10m catheter against the 8mm. During a mean follow up of 19 months the incidence of needing a repeat procedure, due to recurrence, was higher among patients with irrigated tip and 8mm tip as compared to the 10mm-tip. Few studies demonstrated this difference [10,13], most of them considered only a short term follow up.

The limitations of this study reside on the fact that it is a single center registry (cohort) and the comparison is not randomized. We also did not evaluate the effect of the local anatomy of the isthmus on the success of ablation. A previous published paper showed that for patients with a pouched isthmus the irrigated tip gives a better result regarding procedure success [10]. However, this was unlikely a significant issue in our population because bidirectional block was achieved uniformly with all the techniques. The difference in the number of cases ablated with the different catheters should be pointed, as well as, as a potential bias. Also, we need to stress the 'time' bias (cases in the past were done using one type technology that evolved during time to another one). There is no exact explanation for the higher use of fluoroscopy in cases that were done using 3D mapping. The literature on this subject [14] show opposite results. May be the use of the noncontact mapping (9 french - ESI™ balloon / EnSite Array™) in the early cases is responsible for this finding.

Recently, a new type of irrigated catheter with more ports for active cooling on the ablation tip has been introduced on the market, but there is no clinical study comparing this type of catheter with others. Also the use of cryoablation with a large tip has been demonstrated to be effective for AFL ablation, but its results were inferior to RF using a 8mm catheter [15].

In our series we found a high rate of acute success with the use of different techniques for AFL ablation. The procedure, fluoroscopic and total radiofrequency delivery times were shorter with the use of 10mm tip as compared with the others techniques. The long-term risk of recurrence was lower when we using the 10mm-tip catheter and the survival free of a second procedure was higher among patients treated with this catheter. Further randomized trials are required to confirm these findings.

## Figures and Tables

**Table 1:**
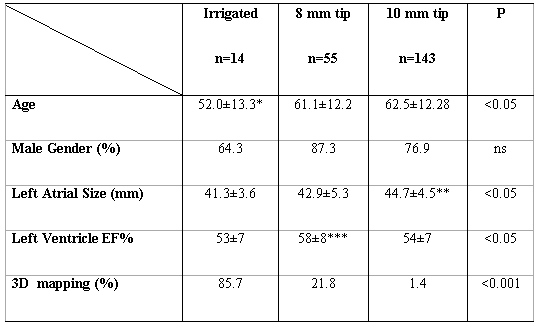
Clinical characteristics of all patients

* difference among irrigated vs. 8mm and vs. 10mm; ** difference among 10mm vs. 8mm and vs. irrigated; *** difference among 8mm vs. 10mm. ANOVA with post-hoc analisys using Student-Newman-Keuls test for all pairwise comparisons.
